# Notes and News

**Published:** 1895-08-17

**Authors:** 


### Notes and News.

(Collected and Communicated.)

At the annual meeting of the Sheffield Royal Hospital it was reported that the first portion of the new building had been opened, and that the financial position of the charity had improved. An alteration of the rules was made to the effect that the institution should in future be called the Sheffield Royal Hospital, instead of the Sheffield Hospital and Dispensary as heretofore. The Duke of Norfolk was re-elected president.

At the annual meeting of the Amcoats Hospital, held on July 31st, both the reader of the report and the speakers who followed insisted on the necessity of supplementing the work of the hospital by increased provision of convalescent homes. After that period of illness was passed in which purely hospital treatment was necessary, a time came in which strength returned but slowly unless patients could be removed into the country or to the health-giving breezes of the sea.

The annual congress of the British Institute of Public Health commenced its session at Hull on August 8th, under the presidency of the Mayor, Alderman Richardson, who, in welcoming the members present, described various matters of sanitary interest in the locality. Much time, thought, and money had been expended upon sanitary matters, not only to protect the inhabitants of the port, but also the dwellers in the inland centres of population, from any infectious disease that might be brought to our shores. The Sheriff of Hull and the Mayors of several other seaboard towns pointed out the expense to which they had been put in providing means for preventing an invasion of cholera, and considered that this expense ought to be a national charge, and should not be borne entirely by the seaports. Addresses have on succeeding days been delivered by Sir A. K. Rollit, M.P., Professor Smith, and others, and the usual festive side of such congresses has not been either forgotten or neglected.

A Congress of Hygiene will be held at Bordeaux in November on the occasion of the exhibition to be held there.

The spread of small-pox in London has led the managers of the Metropolitan Asylums Board to re-open the Gore Farm Hospital for small-pox convalescents. With this object the admission of scarlet fever convalescents has been stopped, and those already in the buildings have been removed. The fact that cases of small-pox have been reported from twenty-six out of the thirty-two poor-law districts shows the wide-spread nature of the present outbreak. Unfortunately, the closing of Gore Farm against convalescent fever cases has seriously crippled the Board in their endeavours to deal effectually with scarlet fever, it now being again this year quite impossible to receive all even of the urgent cases for whose admission to hospital application has been made.

At a special meeting of the Belfast Royal Hospital, held on August 5th, it was resolved to spend, out of invested capital, a sum not exceeding £1,500 on sanitary and other improvements which are urgently required. It seems quite clear that there has been much room for improvement in the arrangements of this hospital, and we can only express surprise, tempered by a little doubt, at hearing that the sum of £1,500 will make things satisfactory. The prospect of some time or other removing to more commodious premises is apt at all times to have an injurious influence on hospital administration, tempting managers to put up with conditions which they certainly would not accept as a permanency. Such prospects, however, are but small solace to the patients of the immediate present. We find it difficult to reconcile the extreme emphasis laid upon the necessity of economy, which had, apparently, led to the cutting down of improvements estimated to cost £4,000 to such as can be carried out for £1,200, with the statement made at the meeting that while ten years ago the invested funds amounted to £18,000 they are now £40,000.

The Grosvenor Hospital for Women and Children is issuing an urgent appeal for £3,000 to complete the sum necessary to finish the new buildings. The work of the hospital has been carried on with difficulty in inadequate premises, but the generous gift of £8,000, presented through a member of the medical staff to the building fund, has now made it possible for the work of reconstruction to be begun in earnest, with the object of erecting a small hospital, containing only a few beds, but in every way up to the requirements of modern science. The estimated total cost of the new buildings is £12,000, and, in addition to the sum mentioned above, £1,000 has been subscribed, thus leaving £3,000 to be obtained. New annual subscriptions are at the same time much needed to maintain the hospital in efficiency. Contributions will be thankfully received by the secretary at the hospital, or they may be paid direct to the London and County Bank, Westminster branch, Victoria Street. The late Lord Shaftesbury was warmly interested in the Grosvenor Hospital. Lord Cross is its present president, and Princess Christian, the Duchess of Teck, Baroness Burdett-Coutts, and the Duke of Westminster are among its supporters.

